# The text2term tool to map free-text descriptions of biomedical terms to ontologies

**DOI:** 10.1093/database/baae119

**Published:** 2024-11-28

**Authors:** Rafael S Gonçalves, Jason Payne, Amelia Tan, Carmen Benitez, Jamie Haddock, Robert Gentleman

**Affiliations:** Stanford Center for Biomedical Informatics Research, Stanford University, 3180 Porter Dr, Palo Alto, CA 94304, United States; Center for Computational Biomedicine, Harvard Medical School, 10 Shattuck St, Boston, MA 02115, United States; Department of Biomedical Informatics, Harvard Medical School, 10 Shattuck St, Boston, MA 02115, United States; Department of Mathematics, Harvey Mudd College, 301 Platt Blvd, Claremont, CA 91711, United States; Department of Mathematics, Harvey Mudd College, 301 Platt Blvd, Claremont, CA 91711, United States; Center for Computational Biomedicine, Harvard Medical School, 10 Shattuck St, Boston, MA 02115, United States

## Abstract

There is an ongoing need for scalable tools to aid researchers in both retrospective and prospective standardization of discrete entity types—such as disease names, cell types, or chemicals—that are used in metadata associated with biomedical data. When metadata are not well-structured or precise, the associated data are harder to find and are often burdensome to reuse, analyze, or integrate with other datasets due to the upfront curation effort required to make the data usable—typically through retrospective standardization and cleaning of the (meta)data. With the goal of facilitating the task of standardizing metadata—either in bulk or in a one-by-one fashion, e.g. to support autocompletion of biomedical entities in forms—we have developed an open-source tool called text2term that maps free-text descriptions of biomedical entities to controlled terms in ontologies. The tool is highly configurable and can be used in multiple ways that cater to different users and expertise levels—it is available on Python Package Index and can be used programmatically as any Python package; it can also be used via a command-line interface or via our hosted, graphical user interface–based web application or by deploying a local instance of our interactive application using Docker.

**Database URL**: https://pypi.org/project/text2term

## Introduction

Scientific data repositories typically require data contributors to create metadata that describe their datasets as a way to enhance the discoverability, usefulness, and overall quality of the data. However, there is often insufficient control of the metadata associated with scientific datasets when those data are deposited in data repositories, resulting in poor-quality metadata that hinder the usability of those datasets. For instance, in a study of the quality of metadata in the National Center for Biotechnology Information (NCBI) BioSample [1]—a repository of metadata about biological samples—the authors found substantial variability in the values given by users for key database fields such as “disease” [2], where data authors provide syntactically different strings to denote the same disease or condition (e.g. “cardiac failure,” “heart failure,” and “myocardial failure”). This is the kind of problem that the many controlled vocabularies, medical terminologies, and ontologies are designed to mitigate—e.g. the Unified Medical Language System (UMLS) [3] contains 185 vocabularies and ontologies used in the medical domain; the BioPortal [4] ontology repository contains over 1100 biomedical ontologies. As a result of these quality issues, data are harder to find and are often burdensome to reuse, analyze, or integrate with other datasets due to the upfront curation effort required to make the data usable—typically through retrospective standardization and cleaning of the (meta)data. The broader consequence to science is that the effectiveness of performing biomedical research through reuse of archived data is substantially diminished.

With growing awareness and adoption of the FAIR data principles [5] to make data findable, accessible, interoperable, and reusable, scientists are increasingly attempting to add semantic annotations to their data. Although there are various tools that enable users to annotate free text in their metadata with one or more ontologies, it is unusual to see real-time control of metadata in data submission portals such as autocomplete-type input fields for key metadata elements describing the submitted data that use ontologies or controlled vocabularies as providers of acceptable input values. This could be in part because existing tools are primarily designed to facilitate the “retrospective” standardization of discrete entities and metadata elements, such as names of diseases, phenotypes, cell types, or chemicals.

There are two broad mechanisms for mapping terms to ontologies: programmatically by leveraging dedicated packages, command-line interfaces, or Application Programming Interfaces (APIs) or interactively by leveraging user interface–based tools developed for the purpose. In this paper, we describe the “text2term” tool to facilitate both programmatic and interactive mapping (or “grounding”) of plain-text entity descriptions to controlled terms in ontologies. The tool is open-source, openly developed, and distributed under a Massachusetts Institute of Technology (MIT) license. A Python package—available in the Python Package Index (PyPI) repository of software for the Python programming language—provides the main engine for programmatic mapping. A React JavaScript–based web application provides user interfaces for interactive mapping, which can be deployed on any machine using Docker. The driving design rationale for the tool was to facilitate the annotation of metadata containing thousands of free-text descriptions of phenotypes [under analysis in Genome-Wide Association Studies (GWASs)] with ontologies. The end goal was to enable integration with and search across biobank-scale resources, which are typically annotated with the Experimental Factor Ontology (EFO) [6], and large health insurance databases. EFO is used to annotate phenotypes in the GWAS Catalog database [7]—the NHGRI-EBI Catalog of human GWASs, in the OpenGWAS database [8], and in the OpenTargets platform [9]. We evaluate the effectiveness of text2term based on how accurately it can refind mappings that have been identified and accepted by human experts and are openly available to use and to download. Our benchmark test corpus consists of three mapping sets: the biomappings [10] collection of community-curated and contributed ontology mappings, the ontology mappings hosted in the Ontology Lookup Service (OLS), and the mapping set of UK Biobank phenotypes to EFO [11]. We show that text2term selects the correct mappings with the accuracy of 79%, 81%, and 73% when compared to benchmark mappings, respectively.

## Related work

The BioPortal Annotator [12] is a web service that takes a text input and returns a list of annotations, each consisting of an ontology term and its corresponding location in the text. It can be used through simple web interfaces or via REST APIs. BioPortal Annotator uses a concept recognition system called Mgrep [13] to perform the string-matching between the text and the ontology terms. Mgrep is a fast and scalable string-matching tool that uses a tree-based data structure to store the ontology terms and their synonyms. Mgrep can handle exact and approximate matching, as well as regular expressions and wildcards.

CEDAR [14] develops a tool for metadata management—the CEDAR Workbench [15]—that can be configured to constrain inputs in specific (metadata) form fields to terms in one or more ontologies or specific branches of ontologies. When a user is filling in a metadata form, CEDAR uses the BioPortal Annotator service to get matches between the user input and ontology terms.

Zooma [16] is a web service that maps free-text annotations to ontology terms based on a curated repository of annotation knowledge. It is designed to help researchers annotate their experimental data with ontology terms The tool uses a combination of curated mappings in existing datasets and standard text matching to map text to ontologies.

SORTA [17] is a system for ontology-based recoding and technical annotation of biomedical phenotype data. It maps input string values to a target ontology using Lucene and N-gram-based matching. Lucene is an open-source text search engine library that allows fast and flexible indexing and querying of text data. SORTA is an open-source system and available as a web service.

The clinical Text Analysis and Knowledge Extraction System (cTAKES) [18] is an open-source tool for mapping free-text descriptions of biomedical entities to ontology terms. It uses Apache’s Unstructured Information Management Architecture framework and the OpenNLP toolkit to create linguistic and semantic annotations of clinical texts at scale.

MARIE [19] is an unsupervised learning-based tool designed to find controlled terms for input strings. MARIE employs a unique combination of string-matching methods and term embedding vectors generated by BioBERT. This approach allows the tool to utilize both structural and contextual information to calculate similarity measures between source and target terms. (The tool could not be executed at the time of writing and is not actively maintained.)

MetaMap [20] is a mapping tool developed by the National Library of Medicine that provides access to the concepts in the UMLS Metathesaurus. The mapping process begins with lexical/syntactic analysis, which includes tokenization, sentence boundary determination, acronym/abbreviation identification, and part-of-speech tagging. MetaMap’s string-matching algorithm links the processed text to the knowledge embedded in the Metathesaurus, including synonym-type relationships.

Overall, interactive mapping is generally not well supported in the current landscape of tools. Some of the tools, such as SORTA or cTAKES, include user interfaces that present the mapping or annotation results; however, none of them support editing the mappings in place, nor do they provide in-place visual context of the mappings (e.g. the neighboring ontology terms of each mapping). Programmatic mapping is also not well supported, with only BioPortal and Zooma providing adequate interfaces for this purpose. However, the use of these services relies on internet access, and often the services impose measures to curb requests in bulk by throttling them, which results in slow response time when mapping many terms Overall, there currently is no one solution that provides both easy programmatic operation and user interface support for visualizing, verifying, and editing the generated mappings.

## The “text2term” tool

**Figure 1.**
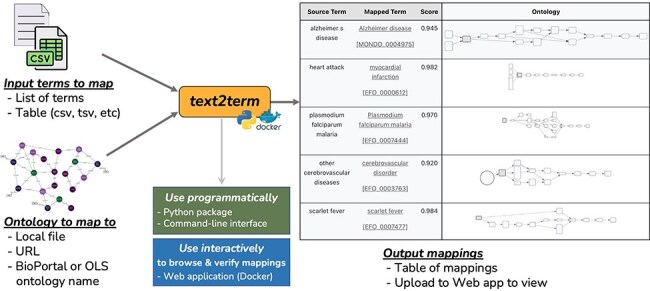
Overview of the text2term tool to map input terms (provided as a list of strings or as a table) to an ontology (specified by a file path, URL, or the ontology name as used by BioPortal or OLS for their Annotator and Zooma services).

The text2term mapping tool (Fig. 1) is designed to be a flexible computational instrument to generate potential ontology term mappings for arbitrary strings, using one of several similarity metrics and leveraging ontology details such as synonyms. It was built out of the need to have both programmatic and interactive mechanisms to compute and verify mappings of free-text descriptions of phenotypes in biomedical metadata to one or more Web Ontology Language (OWL) ontologies.

### Tool design and features

The text2term tool provides support for (i) popular edit distance metrics such as the Levenshtein distance commonly used in spell checkers; (ii) an approach that is based on vectors computed using Term Frequency-Inverse Document Frequency (TF-IDF), which is a popular metric used in information retrieval; (iii) an interface to the BioPortal Annotator tool, which allows mapping to any ontology in the BioPortal repository—the largest repository of biomedical ontologies; and (iv) an interface to the Zooma Annotator tool, which allows mapping to any ontology in the OLS repository of biomedical ontologies. The text2term tool can be used in two ways: (I) “programmatically” by importing the text2term package from a Python environment or by using a command-line interface and (II) “interactively” via a web application with user interfaces for input entry, mapping visualization, verification, and download. The web application can be deployed locally using Docker, which is the ideal way to support bulk or repetitive mapping jobs; it can also be used directly on our hosted version at https://text2term.hms.harvard.edu, which is practical for smaller jobs.

Once installed, the text2term package is available to be imported from Python environments and used programmatically. The tool is also executable from the command line, which does not require any programming. Multiple configuration options, described next, allow users to fine-tune text2term both programmatically and through the command line. The documentation of the tool is provided on GitHub as both a conventional README and a GitHub Page (https://rsgoncalves.github.io/text2term) and in the “readthedocs” platform—a widely used, open-source software documentation hosting platform (https://text2term.readthedocs.io). Additional details about the tool are provided in the Supplementary Material, along with a complete list of features in Supplementary Table S1.

#### Input terms to map

text2term takes as input a list of strings or a file containing strings to be mapped, which we call “source terms.” The file input can be either a text file containing a line-separated list of terms or a table file where cells are delimited by some character (such as CSV or TSV) that can be configured via an argument in text2term.

#### Ontology input and processing

text2term requires a “target ontology,” which can be any ontology specified in the W3C standard OWL format, the *de facto* ontology language used nowadays. The target ontology can be provided as a local file path or as a URL to an ontology resource that can be resolved using standard URL handling methods—in our case, we use Python’s built-in “urllib” URL handling modules. Furthermore, text2term can also work with ontologies hosted in the popular BioPortal and OLS repositories of biomedical ontologies.

We use the “owlready2” [21] library to load ontologies, to perform ontology reasoning, and to obtain ontology term details such as labels, synonyms, and definitions. Specifically, the tool collects the following details for each ontology term:

- Human-readable labels specified via “rdfs:label” or “skos:prefLabel” relationships.
- Synonyms specified via the “obo:hasExactSynonym” relationship, commonly used across biomedical ontologies such as the Monarch Disease Ontology (MONDO) [22], or via the ontology-specific synonym relationships in the NCI Thesaurus (NCIT:P90) and in the EFO (EFO:alternative_term). Optionally, broad synonyms (obo:hasBroadSynonym) can be included.
- Definitions specified via skos:definition or the Information Artifact Ontology (IAO) definition property (IAO:0000115).
- Hierarchical (rdfs:SubClassOf) relationships between ontology terms; specifically, we collect the parents, children, and instances of each ontology term. This can be done before or after reasoning, depending on the user configuration—by default, reasoning is not performed.

The ontology term details collected in Steps 1–3 are used to match the input strings with ontology terms, while currently, the details from Step 4 are used for visualization purposes.

#### Matching methods

The text2term tool is designed to support, in a pluggable way, multiple string-matching techniques that users can select from. We mostly reuse existing methods and implementations available for string comparison. However, because of the poor performance we initially observed using these methods, we decided to design our own, simple approach that converts strings to vectors (using TF-IDF) and operates based on vector (cosine) similarity.

##### TF-IDF-based mapper

TF-IDF is a statistical measure often used in information retrieval and text mining—essentially, it measures how important a token is to a document relative to its occurrence across a corpus of documents. In our case, we consider as documents the terms in an ontology—specifically, the labels and exact synonyms of ontology terms. We obtain TF-IDF-based vectors for the input query strings and ontology terms, and then we use the widely used cosine similarity measure to compare vectors, which is the cosine of the angle between two vectors. In our implementation we use the “scikit-learn” library [23] to compute TF-IDF and the “sparse-dot-topn” library [24] to compute cosine similarity between TF-IDF vectors. After experimenting with other implementations of cosine similarity—such as the built-in function in “scikit-learn”—we concluded that the “sparse-dot-topn” implementation provides a faster, more memory efficient way to compute the multiplication of two sparse matrixes and to obtain the top-*n* closest values per query.

##### BioPortal and Zooma Web API-based mappers

We implemented Python interfaces to obtain mappings from two popular web services for annotating unstructured text with ontology terms—the BioPortal Annotator and the Zooma Annotator in OLS—through their respective web/REST APIs. Our BioPortal mapping interface enables programmatically annotating terms with any ontology in the BioPortal repository by specifying the ontology name as used in BioPortal. Similarly, our Zooma interface allows annotating terms with ontologies available in the OLS repository by using their respective names as used in OLS. Ontology names are often, but not always the same between the two repositories. BioPortal does not return confidence scores for its annotations. We used a mapping score of 1 for all BioPortal annotations.

##### Syntactic distance–based mappers

We also provide support for commonly used and well-known syntactic (edit) distance metrics. Specifically, we implemented support for matching input strings with ontology terms using the Levenshtein, Jaro, Jaro–Winkler, Jaccard, and Indel metrics.

The syntactic distance–based mappers as well as the Web API-based mappers perform slowly, since they do pairwise comparisons between each input string and each ontology term label and/or synonym, and there are networking and API load overheads for the web API-based approaches. The TF-IDF-based approach reduces the mapping problem to matrix operations that are much faster to compute—this makes it possible to process tens of thousands of input queries in <1 min.

### Interactive web application

We developed a web application with user interfaces designed to facilitate the process of specifying tool inputs and to enable interactive curation of the mappings generated by text2term. The frontend of the web application is implemented using React—a JavaScript library for building user interfaces. In the backend, we use the Flask framework to implement RESTful APIs that allow interfacing with the text2term package, resuming a mapping session, or obtaining mapping results. We provide a Docker image that can be deployed in any machine with two simple commands.

**Figure 2.**
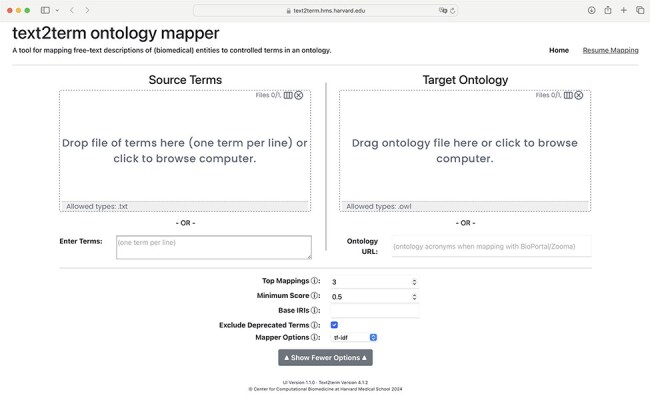
Front page of the text2term Web application (https://text2term.hms.harvard.edu) where users specify input terms by uploading a file or entering raw text, and the target ontology to map those terms to, which can be uploaded or pointed to via its URL.

**Figure 3.**
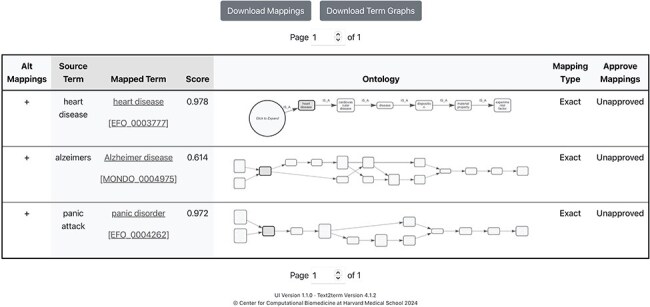
Results page showing mappings generated by text2term where, from left to right, users can see in each row: a button to “view alternate mappings” that shows mappings with lower scores, which users can then choose from; the “Source Term” that was given as input; the top-scored “Mapped Term” for that input and the corresponding mapping score; a graphical depiction of the “neighborhood” of the ontology term, which contains the sub- and superclasses of the term; a user option to specify the mapping type (Exact, Broad, or Narrow—subproperties of the standard “skos:mappingRelation” property defined in the Simple Knowledge Organization System (SKOS) vocabulary); and finally a dropdown menu for users to specify whether the mapping is approved.

**Figure 4.**
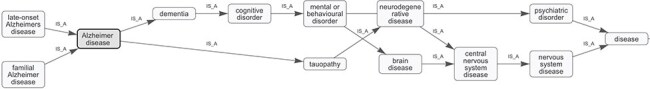
Ontology graph visualization provided by the text2term web application, showing all ancestors of a term, in this case “Alzheimer disease,” as well as direct subclasses.

The front page of the application is shown in Fig. 2, where users specify, at a minimum, the input “source” terms, and the “target” ontology to map the terms to. Some additional, basic options are included in the user interface (UI) to allow users to configure the tool. It is possible to resume a mapping session by clicking on the “Resume Mapping” link and then uploading a previously downloaded mapping table. The tool will effectively display the mapping results table marked up just as it was upon downloading. After submitting source terms and target ontology for mapping, users are presented with a table of mapping results, as shown in Fig. 3. The mapping results contain the input source terms, their top-ranked mapped terms in the ontology, and then a visualization (shown in more detail in Fig. 4) of where in the ontology class hierarchy those mapped terms are located.

The base functionality for the interface is the same as that of the programmatic tool described earlier. However, it should be noted that there are several features and options in the programmatic tool that are not available on the interface. This includes (but is not limited to) features such as caching, term-type specification, and unmapped terms in the output.

### Usage metrics

**Figure 5.**
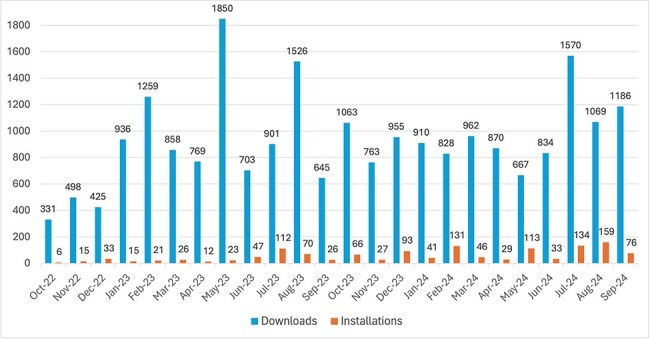
Number of downloads from PyPI and of installations via pip (*y*-axis) per month since the first release on 20 October 2022 until 30 September 2024 (*x*-axis).

The text2term tool can be easily leveraged programmatically from a Python environment. After installation, there is a single function called “map_terms” that allows users to perform mapping. Since its inception in October 2022, the text2term Python package has been downloaded 22 378 times from PyPI. (We obtain download counts by querying the public PyPI download statistics dataset using Google BigQuery. Our queries are specified and documented in our evaluation repository for replicability: https://github.com/rsgoncalves/text2term-evaluation.) On average, text2term is downloaded 973 times per month (Fig. 5). The text2term Python package has been installed via the “pip” installer a total of 1354 times, and on average, it is installed 59 times per month (Fig. 5).

The text2term web application hosted in our publicly accessible server has been lightly but regularly used since it first went live in August 2022. To date, our server has processed just over 400 requests—on average, it receives about 20 requests per month.

## Comparison with verified mappings

In this section, we describe a simple comparison between the mappings generated by our text2term tool and publicly available mappings verified by human curators. The initial motivation for the development of our tool was to facilitate bulk mapping of free-text descriptions of phenotypes to controlled terms in ontologies. As such, we attempted to identify ground-truth “benchmark” datasets that mimic precisely such a task of mapping phenotype descriptions to ontology terms.

## Methods and materials

We identified a mapping set—the EFO-UK Biobank (UKB) mappings [11]—where the authors undertake the same task as we set out to do. We then add to our test corpus two generic collections of mappings between controlled terms in ontologies, controlled vocabularies, etc. Our test corpus consists of the three public mapping sets described in the following:

- EFO-UKB mappings—a collection of mappings between phenotype descriptions in the UK Biobank and terms in the EFO ontology. Some of the phenotypes in the UK Biobank—a widely used source of population health data in research—are mapped to the 10th International Classification of Diseases (ICD-10). However, ICD-10 codes are not exhaustively mapped to public ontologies, which hinders interoperability of UK Biobank with public data. The authors built this mapping set using the Zooma ontology mapping tool followed by manual curation of mappings.
- OLS mappings—a collection of mappings between biomedical ontologies that are hosted in the OLS repository [25]. These mappings are extracted directly from the ontologies, which have been specified by the respective ontology engineers. The OLS mappings are specified in the Simple Standard for Sharing Ontology Mappings (SSSOM) format [26].
- Biomappings—a collection of community-contributed mappings between biomedical entities [10], which are not available from primary sources (such as OLS). The goal of this mapping collection is ultimately to be integrated with primary mapping sources and be distributed among other established mappings. Biomappings are also distributed in SSSOM format.

These mapping sets represent reasonable benchmarks and opportunities to assess the quality of our tool-generated mappings, since they have been verified by human experts and are useful public mapping sets that are either widely used or have the potential to be widely used.

Our comparison is limited to mappings to the EFO ontology, although the text2term tool is entirely generic and can be used with arbitrary OWL ontologies (or with ontologies hosted in BioPortal and OLS). We also limit our comparison to queries that have exactly one ontology mapping in the benchmark mapping set, since such a singular mapping is unambiguously indicative that one and only one term was the appropriate choice to annotate the given input. We use the TF-IDF-based mapper in text2term, as we observed that it is significantly faster and more effective than either edit distance-based mappers or the Web API-based approaches.

**Figure 6.**
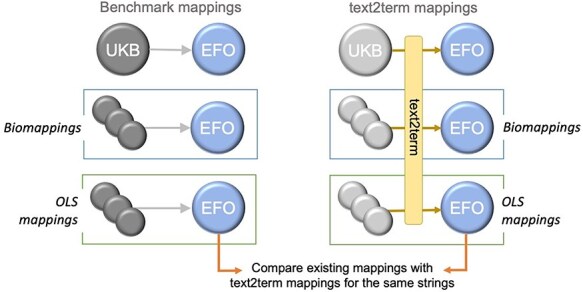
Overview of the design of our comparison between existing and text2term-generated mappings for the same text inputs.

We set out to compare these three mapping sets—our “benchmark mappings”—with the mappings generated by text2term for the same inputs, as depicted in Fig. 6.

Specifically, we want to determine whether text2term mappings coincide with the human-verified ones, whether they are more specific or more general (according to the ontology class hierarchy), whether they are siblings (i.e. they have a common direct parent), or whether they are (hierarchically) unrelated. In the case of the biomappings and OLS mappings, we use the label of each “source” ontology term as the free-text input.

### Mapping categorization

Consider a tool-generated mapping *T* and a human-verified mapping *H* for some input text *I*. We categorize *T* as follows:

- Same: if *T* is the same as *H*.
- More specific: if *T* is a subclass of *H*, i.e. EFO entails *T* SubClassOf *H*.
- More general: if *T* is a superclass of *H*, i.e. EFO entails *H*SubClassOf *T*.
- Sibling: if *T* and *H* have the same direct superclass, i.e. EFO states that *T* SubClassOf *P* and *H* SubClassOf *P*.
- Unrelated: if there is no sub- or superclass relation between *T* and *H*.

We compute mappings of the (label) strings contained in each benchmark mapping set to the EFO ontology v3.62.0, released on 15 January 2024 (https://github.com/EBISPOT/efo/releases/tag/v3.62.0). We use text2term v4.1.2, released on 6 March 2024. All benchmark mapping sets and mappings computed by text2term in our comparison, along with the code to reproduce the entire comparison, are hosted in a GitHub repository at https://github.com/rsgoncalves/text2term-evaluation.

## Results

The results of the comparison of the mappings generated by text2term with those in our benchmark mapping sets are presented in Table 1.

**Table 1.** Results of comparison between mappings generated by text2term and mappings in our selected benchmark mapping sets

Category	UKB-EFO	Biomappings	OLS
Same	660 (73.3%)	626 (78.7%)	6588 (80.9%)
More Specific	34 (4%)	0 (0%)	91 (1.1%)
More General	20 (2.2%)	2 (0.3%)	55 (0.7%)
Sibling	13 (1.4%)	47 (5.9%)	89 (1.1%)
Unrelated	172 (19.1%)	120 (15.1%)	1320 (16.2%)

Overall, we observed that text2term finds (or refinds) mappings with high accuracy in all our selected benchmark mapping sets. We think of this accuracy as a proxy for alleviated human effort, in the sense that, in a scenario where a curator is mapping a collection of strings, on average for some three out of four inputs, text2term picks the correct ontology mapping which the curator simply needs to confirm. In the remaining one out of four inputs, a curator may have to inspect additional potential mappings (remember, users can specify the number of mappings per input string) or resort to other resources.

We now look in more detail at the mappings where, for a given input entity, text2term did not find the expected benchmark mapping. We picked three examples in each category, and in each mapping set, to illustrate some patterns that we observe across many of the other mappings. The selected mapping pairs are shown in Tables 2–4.

**Table 2.** Example mappings selected from the results of the comparison of text2term with the UKB-EFO mappings

Input entity	text2term mapping	Benchmark mapping	Category
Granulomatous disorders of skin and subcutaneous tissue	Skin disease (EFO:000070)	Granulomatous dermatitis (EFO:1000705)	More General
Hayfever or allergic rhinitis	Allergic rhinitis (EFO:0005854)	Seasonal allergic rhinitis (EFO:0003956)	More General
General pain for 3+ months	Pain (EFO:0003843)	Chronic pain (HP:0012532)	More General
Uremia	Uremia (EFO:1001226	Kidney failure (EFO:1002048)	More Specific
Thyroiditis	Thyroiditis (MONDO:0004126)	Thyroid disease (EFO:1000627)	More Specific
Thyroid problem (not cancer)	Thyroid cancer (MONDO:0002108)	Thyroid disease (EFO:1000 627)	More Specific
Gynecomastia	Gynecomastia (HP:0000771)	Breast hypertrophy (HP:0010313)	Sibling
Emphysema/chronic bronchitis	Chronic bronchitis (EFO:0006505)	Emphysema (EFO:0000464)	Sibling
Bursitis	Bursitis (MONDO:0002471)	Frozen shoulder (EFO:1000 941)	Sibling
Alzheimer’s disease	Alzheimer disease (MONDO:0004975)	Alzheimer’s disease (EFO:0000249)	Unrelated
Senile cataract	Senile cataract (MONDO:0004847)	Age-related cataract (HP:0011141)	Unrelated
*Helicobacter pylori*	*Helicobacter pylori* infectious disease (EFO:1000961)	*Helicobacter pylori* (NCBITaxon:210)	Unrelated

**Table 3.** Example mappings selected from the results of the comparison of text2term with Biomappings, where entities with “DOID”or “D” at the start of their identifier are terms from the Human Disease Ontology (DOID), while entities with “HP” in their identitier are from the Human Phenotype Ontology (HP)

Input entity	text2term mapping	Benchmark mapping	Category
Staphylococcal Infections (D013203)	Staphylococcal skin infections (EFO:1001849)	*Staphylococcus aureus* infection (EFO:0005681)	More General
Venous Thrombosis (D020246)	Venous thrombosis (HP:0004936)	Deep vein thrombosis (EFO:0003907)	More General
Autoimmune disease (DOID:417)	Type II hypersensitivity reaction disease (EFO:0005809)	Autoimmune disease (EFO:0005140)	Sibling
Non-ST Elevated Myocardial Infarction (D000072658)	ST Elevation Myocardial Infarction (EFO:0008585)	Non-ST Elevation Myocardial Infarction (EFO:0008586)	Sibling
Calu-3 (CALU3_LUNG)	Calu1 (EFO:0002151)	Calu3 (EFO:0002819)	Sibling
Childhood-onset asthma (DOID:0080815)	Childhood onset asthma (MONDO:0005405)	Childhood onset asthma (EFO:0004591)	Unrelated
Muscular Atrophy (D009133)	Skeletal muscle atrophy (HP:0003202)	Muscle atrophy (EFO:0009851)	Unrelated
Neuralgia (D009437)	Neuralgia (EFO:0009430)	Neuropathic pain (EFO:0005762)	Unrelated

**Table 4.** Example mappings selected from the results of the comparison of text2term with the OLS mappings

Input entity	text2term mapping	Benchmark mapping	Category
Astrocytic Tumor (NCIT:C6958)	Astrocytic tumor (MONDO:0021636)	Astrocytoma (EFO:0000272)	More General
Meningitis (DOID:9471)	Meningitis (MONDO:0021108)	Infectious meningitis (EFO:0000584)	More General
Carcinoma de tiroides (HP:0002890)	Carcinoma (EFO:0000313)	Thyroid carcinoma (EFO:0002892)	More General
Cellular schwannoma (DOID:3196)	Cellular schwannoma (MONDO:0002548)	Schwannoma (EFO:0000693)	More Specific
Brain cancer (DOID:1319)	Brain cancer (MONDO:0001657)	Brain neoplasm (EFO:0003833)	More Specific
Dandy–Walker malformation (NCIT:C75012)	Isolated Dandy–Walker malformation with hydrocephalus (MONDO:0017110)	Dandy–Walker syndrome (EFO:1000890)	More Specific
Stroke disorder (MONDO:0005098)	Stroke disorder (MONDO:0005098)	Stroke (EFO:0000712)	Sibling
Melancholic depression (DOID:1595)	Melancholia (EFO:1002014)	Unipolar depression (EFO:0003761)	Sibling
Polyarteritis nodosa (DOID:9810)	Polyarteritis nodosa (MONDO:0019170)	Polyarteritis Nodosa (EFO:0009012)	Sibling
Vein (XAO:0000115)	Vein disorder (MONDO:0004634)	obsolete_vein (EFO:0000816)	Unrelated
Kaposi’s sarcoma cell (BTO:0002071)	Kaposi’s sarcoma (EFO:0000558)	Kaposi’s sarcoma cell (EFO:0000187)	Unrelated
Tuberculosis (DOID:399)	Tuberculosis (MONDO:0018076)	obsolete_tuberculosis (EFO:0000774)	Unrelated

In Table 2, we see that some mappings selected by text2term are more appropriate for the input entities than the benchmark mappings—e.g. “uremia,” “thyroiditis,” “Gynecomastia,” “bursitis,” and “Senile cataract.” Some other mappings provided by text2term can be considered just as correct as the benchmark mappings, e.g. in the cases of the mappings for the inputs “hayfever or allergic rhinitis,” “emphysema/chronic bronchitis,” or “General pain for 3+ months.” In the Unrelated category, we observe that some benchmark mappings point to deprecated terms—e.g. “Alzheimer’s disease” (EFO:0000249) has been replaced (in EFO) by the term “Alzheimer disease” (MONDO:0004975), which text2term correctly identifies.

In Table 3, there are only two mappings considered more general than the ones in biomappings—text2term correctly identifies more appropriate terms in both cases. In the three selected mappings (out of 47) in the Sibling category, the text2term mappings seem mostly incorrect when compared to biomappings—this happens primarily because of numeric symbols or some form of negation in an input entity text (e.g. “non-”). In the Unrelated category, we start with another example of a benchmark mapping involving a deprecated term [“childhood onset asthma” (EFO:0004591)]. In the other example mappings of the Unrelated category, the text2term recommendations seem just as reasonable as the benchmark mappings—and this highlights perhaps some repetition in EFO due to reuse of terms from other ontologies.

In Table 4, we see once again examples of mappings, in both the More General and the More Specific categories, where text2term identified just as correct terms compared to the benchmark mappings (e.g. “Astrocytic tumor” and “brain cancer”), some times more appropriate terms (e.g. “meningitis,” “cellular schwannoma,” and “stroke disorder”), and other times not as suitable terms (e.g. “Carcinoma de tiroides and “Dandy–Walker Malformation”). In the Sibling category, text2term tends to find more appropriate terms than those in the benchmark mappings—e.g. “stroke disorder” and “melancholic depression,” which are more appropriately mapped to melancholia than to unipolar depression. The last example mappings for “polyarteritis nodosa” reveal again some conceptual duplication within EFO, which contains two non-deprecated terms that unambiguously are intended to describe the same condition. Finally, most of the mappings in the Unrelated category in the OLS mappings are related to obsolete terms in EFO, some of which have been replaced by terms that text2term correctly maps the input entity to—e.g. “vein” and “tuberculosis.”

We next present in Table 5 the accuracy and efficiency of each mapper supported by text2term on the UKB-EFO mapping set. Our comparison includes two popular ontology mapping services commonly used for mapping free-text term descriptions to ontology terms: the BioPortal Annotator and the Zooma tool (discussed in the Related Work section). Other related tools could not be included in our comparison due to technical limitations or issues: MetaMap and cTAKES only work with ontologies stored in UMLS and do not provide support for accepting as input a user-provided ontology in standard OWL format. We could not test these tools on our EFO mapping sets since EFO is not contained in UMLS. The MARIE tool could not be executed using the code (and instructions) provided in the tool’s repository. It also requires external data that could not be obtained. The SORTA tool can be successfully deployed locally using Docker: however, the ontology import wizard throws an error when importing ontologies in both OWL and OBO format, which the tool purports to support. The tool was tested with EFO and small ontologies, all resulting in the same generic error that simply states that an error occurred.

**Table 5.** Performance and effectiveness of mappers included in text2term on the UKB-EFO mapping set

Mapping category	Bioportal	Zooma	TF-IDF	Indel	Jaro	Jaro–Winkler	Jaccard	Levenshtein
Same	330 (42.7%)	583 (65.3%)	660 (73.3%)	542 (60.3%)	495 (55.1%)	452 (50.3%)	332 (36.9%)	524 (58.3%)
More Specific	13 (1.7%)	55 (6.2%)	34 (4%)	40 (4.4%)	26 (2.9%)	22 (2.4%)	18 (2.0%)	40 (4.4%)
More General	154 (19.9%)	18 (2.0%)	20 (2.2%)	24 (2.7%)	26 (2.9%)	33 (3.7%)	9 (1.0%)	21 (2.3%)
Sibling	14 (1.8%)	21 (2.4%)	13 (1.4%)	22 (2.4%)	28 (3.1%)	28 (3.1%)	21 (2.3%)	24 (2.7%)
Unrelated	262 (33.9%)	216 (24.2%)	172 (19.1%)	271 (30.1%)	324 (36.0%)	364 (40.5%)	519 (57.7%)	290 (32.3%)
**Runtime**	2171 s	687 s	4 s	281 s	365 s	372 s	494 s	354 s

Runtime is wall clock time measured using the Python time package.

As shown in Table 5, the TF-IDF-based mapper in text2term performs substantially faster than all other methods tested, and it provides more accurate results on the tested mapping set. The TF-IDF mapper finds the highest number of mappings that are the same as the mappings picked by experts who curated the UKB-EFO mappings. It also finds the least mappings that are unrelated to those picked by experts.

## Discussion and conclusions

The text2term tool is a simple yet versatile open-source tool for mapping free-text descriptions of biomedical entities to controlled terms in ontologies. It is easy to use and can be leveraged in multiple ways to cater to different users and user backgrounds—text2term can be used programmatically as a Python package; via a command-line interface; via our Harvard-hosted, graphical user interface–based web application (https://text2term.hms.harvard.edu); or by deploying a local instance of the web application using our provided Docker image. The tool is versioned and documented on GitHub, where we also keep a public issue tracker.

The text2term tool was developed at the Center for Computational Biomedicine of the Harvard Medical School, and it is actively developed by its creator. It is used at Harvard both for various projects that build data assets for the biomedical community and by a growing external user community. The usage metrics we collected are suggestive of steady demand and usage of our tool. A key design principle behind text2term is to have an extendable collection of mappers, which can be leveraged by users to plug in a different mapping algorithm while taking advantage of the tool’s remaining infrastructure and user interfaces to browse and verify the resulting mappings. By default, text2term supports string comparisons using edit distance-based algorithms such as Levenshtein distance; a TF-IDF-based algorithm that compares strings based on ngrams of configurable size; the BioPortal Annotator web service, which makes it possible to map to any ontology available in the BioPortal ontology repository; or the Zooma Annotator that enables mapping to ontologies in the OLS repository.

Our evaluation of text2term demonstrated that the tool is highly effective at finding appropriate ontology terms to represent input free-text descriptions of biomedical entities. This finding suggests that using such a tool for bulk standardization of free-text entity descriptions can substantially alleviate the amount of human curation needed, since for most inputs, a curator only needs to confirm the mappings generated by the tool. When compared to the (cross-ontology) mappings that are encoded in OLS ontologies or in biomappings, which are contributed and/or reviewed by domain experts, text2term actually exposed some wrong mappings in the current versions of the benchmark mapping sets, as well as some modeling issues in the EFO ontology itself—particularly the existence of duplicate terms to represent the same concept.

The potential applications of text2term are two-fold. First, it can be used to retrospectively standardize free-text entity descriptions in existing data with ontologies. This can be done programmatically using the highly customizable Python package or the command-line interface or interactively using the text2term web application that can be deployed using Docker. Second, text2term can be used to prospectively control the input of terms as part of an autocomplete mechanism—e.g. in a (web) form—that checks user input against ontology terms and displays the most appropriate term options. In this way, all inputs to specific fields that should be grounded with ontologies (e.g. disease or organism names, cell types, etc.) are guaranteed to be controlled, resolvable terms from one or more ontologies of choice.

Nowadays, with advances in large language models (LLMs) such as Generative Pre-trained Transformer (GPT) and Llama, it may appear that the problem tackled in this paper could be solved using LLMs However, in our experiments attempting to instrument GPT-4 and Llama 3 for mapping free-text term descriptions to ontology terms, the results were not encouraging. Often, the LLM suggested an appropriate label for the term, but not always did it pick the expected label for the term in the target ontology. For example, for the input “heart attack,” both LLMs suggest “heart attack” as a label. However, the EFO term for heart attack has as label “myocardial infarction.” The text2term tools easily get this right as they understand ontology relationships and leverage synonyms in its search. Then, to complicate matters, the ontology term identifiers suggested by the LLMs nearly always corresponded to completely different terms, unrelated to the predicted labels. Toro *et al*. [27] also identified this issue, noting that “Ontology terms are typically referred to using non-semantic numeric identifiers (for example, CL:1001502). These can confound LLMs, which have a tendency to hallucinate identifiers.” It seems essential to have LLM-based tools that are ontology-aware in order to handle the issue of hallucinated term identifiers. We imagine that in the future, such LLM-based tools for mapping free text to ontologies will appear, and when that happens, we intend to compare text2term with them. We also intend to investigate a potential new mapper for text2term that leverages an (open-source) LLM.

In future work, we plan to continue to build and maintain text2term based on requirements from projects at the Harvard Medical School, as well as based on public requests for features and enhancements. For example, while we initially built text2term to map strings to ontology classes, a group of users was interested in mapping to ontology properties in order to standardize the names of relationships between entities in some data. So, we enhanced the tool to allow users to specify whether they wish to map to ontology classes, properties, or both. An additional future work direction for us is to more precisely map terms that include some form of negation—in its current form, the tool favors, e.g. an ontology term such as “hodgkin’s lymphoma” for an input string such as “non-hodgkin’s lymphoma,” potentially because “non” is considered a stop word (and thus removed) by one of the libraries that we reuse. The output of the text2term package will be aligned with the required input format of our web application—since they are currently incompatible—so that users can upload programmatically generated mappings to the web application and then verify those mappings through the user interfaces.

In a future version of the text2term web application, we plan to implement new features to allow users to select from a predefined list of popular ontologies to map to—leveraging the existing ontology caching feature. With this enhancement, users will be able to select multiple ontologies to map to, which currently is not possible. We plan to enhance the UI to allow users to download the ontology graph as an image, to select potentially multiple mappings for each input term, and to attach free-text notes to each mapping. We also plan to enhance the web application to avail of all the configuration options in the Python package, such as including unmapped terms or specifying as input a table containing terms to map. Finally, we intend to implement multiple UI/User Experience improvements, such as more intuitive presentation and selection of alternative mappings (e.g. via a specific button to select an alternative mapping), and more streamlined approval of mappings using buttons rather than a dropdown list. We intend to make both the web application and the Python package more robust, by improving testing coverage, error handling, and better presentation of errors in the UI of the web application.

## Supplementary Material

baae119_Supp

## Data Availability

The text2term tool is freely available, open-source, and distributed under the MIT License. The backend Python package is hosted and versioned on GitHub (https://github.com/rsgoncalves/text2term) and distributed via PyPI (https://pypi.org/project/text2term). The frontend web application codebase is also hosted and versioned on GitHub (https://github.com/rsgoncalves/text2term-ui). The application can be deployed in local systems using Docker, following the instructions provided in the GitHub repository. A fully functional instance of the web application is available for public use in our server at https://text2term.hms.harvard.edu. For large jobs or to avoid network latency, we recommend using the Docker installation or the Python package. The code to reproduce the evaluation and the resulting mapping comparison data can be found at https://github.com/rsgoncalves/text2term-evaluation.
