# Supplementary material for: The text2term tool to map free-text descriptions of biomedical terms to ontologies
Source: Database (Oxford). 2024 Nov 28;2024:baae119. doi: 10.1093/database/baae119 (PMC11604108; doi:10.1093/database/baae119)
Supplement: baae119_Supp [file baae119_supp.zip › suppl_data/TableS1.docx]

| **Option name** | **Type** | **Description** |
| --- | --- | --- |
| **source_terms*** | string/list/dictionary | One of the following: 1) a string that specifies a path to a file containing the terms to be mapped, 2) a list of the terms to be mapped, or 3) a dictionary where each key is a term to be mapped, and each value is a list of tags |
| **target_ontology*** | *string* | Path or URL or acronym of 'target' ontology to map the source terms to. When the chosen mapper is BioPortal or Zooma, provide a comma-separated list of ontology acronyms (eg 'EFO,HPO') or write 'all' to search all ontologies. When the target ontology has been previously cached, provide the ontology name that was used to cache it. As of version 2.3.0, it is possible to specify ontology acronyms as the target_ontology (eg "EFO" or "CL"), which is achieved using bioregistry to retrieve URLs for those acronyms |
| **base_iris** | *tuple* | Map only to ontology terms whose IRIs start with one of the strings given in a tuple, for example: ('http://www.ebi.ac.uk/efo','http://purl.obolibrary.org/obo/HP') |
| **csv_column** | *tuple* | Specify a column to map if a CSV is the input file. Ignored otherwise. |
| **source_terms_ids** | *tuple* | Collection of identifiers for the given source terms |
| **excl_deprecated** | *boolean* | Exclude ontology terms stated as deprecated via *owl:deprecated true* |
| **mapper** | *Mapper* | Method used to compare source terms with ontology terms. One of: levenshtein, jaro, jarowinkler, jaccard, indel, tfidf, zooma, bioportal |
| **max_mappings** | *int* | Maximum number of mappings returned per source term |
| **min_score** | *float* | Minimum similarity score [0,1] for the mappings (1=exact match) |
| **output_file** | *string* | Path to the output file for mappings |
| **save_graphs** | *boolean* | Save graphs representing the neighborhood of each ontology term |
| **save_mappings** | *boolean* | Save the generated mappings to a file (specified by output_file) |
| **separator** | *string* | Character that separates source term values in a character-separated file input. Ignored otherwise |
| **use_cache** | *boolean* | Use a previously cached ontology |
| **term_type** | *Ontology*  *TermType* | Specifies whether to map to ontology classes, properties, or both |
| **incl_unmapped** | *boolean* | Include all unmapped terms in the output, even if they are scored below the min_score |
